# Expected Mitochondrial Haplotype Richness in Remaining Populations of the Critically Endangered European Mink *Mustela lutreola* and Its Conservation Implications

**DOI:** 10.3390/ijms26209935

**Published:** 2025-10-12

**Authors:** Jakub Skorupski, Przemysław Śmietana, Christian Seebass, Wolfgang Festl, Alexe Vasile, Natalia Kiseleva, Florian Brandes, Mihai Marinov

**Affiliations:** 1 Institute of Marine and Environmental Sciences, University of Szczecin, Wąska 13 St., 71-415 Szczecin, Poland; 2 Polish Society for Conservation Genetics LUTREOLA, Maciejkowa 21 St., 71-784 Szczecin, Poland; 3 EuroNerz e.V., Kleine Gildewart 3 St., 49074 Osnabrück, Germany; 4 The “Danube Delta” National Institute for Research and Development, Babadag 165 St., 820112 Tulcea, Romania; 5 Ilmen State Nature Reserve SU FRC MG UB RAS, Chelyabinsk District, Ilmen Reserve, 456317 Miass, Russia; 6 Wildtier-und Artenschutzstation e.V., Hohe Warte 1 St., 31553 Sachsenhagen, Germany

**Keywords:** conservation genetics, European mink, haplotype richness, mitogenome, *Mustela lutreola*, mtDNA, negative binomial model, non-parametric estimators, extrapolation, rarefaction

## Abstract

The European mink *Mustela lutreola* is one of the most threatened carnivores in Europe, having suffered dramatic range contractions and severe population fragmentation. Accurate knowledge of its genetic diversity is crucial for conservation planning, yet earlier studies based on partial mitochondrial markers offered limited resolution and often underestimated haplotype richness. In this study, complete mitochondrial genomes from four extant populations (Russia, *n* = 11; Romania, *n* = 16; Germany, *n* = 24; France–Spain, *n* = 15) were analysed using a suite of non-parametric and asymptotic estimators (Fisher’s *α*, *ACE*, *Jackknife1*, *Bootstrap*, *Chao1*-based iNEXT) together with negative binomial modelling. A total of 41 haplotypes were detected, but extrapolated estimates indicated substantially higher richness, particularly in populations dominated by singletons. Rarefaction and extrapolation analyses revealed that sample sizes of 70–130 individuals per population are needed to approach complete haplotype detection. The France–Spain and Romania populations harboured the highest predicted richness, whereas Germany and Russia, both represented by ex situ stocks, showed lower diversity. These results refine earlier assumptions of extreme homogeneity in the Western population and demonstrate that significant mitochondrial variation persists at the continental scale. The study provides quantitative benchmarks for sampling design and genetic management, supporting preservation of evolutionary potential in this critically endangered species.

## 1. Introduction

A fundamental prerequisite for undertaking effective and well-targeted efforts aimed at the conservation of gene pools of endangered populations or entire species is a proper diagnosis of the causes of their decline [1]. Such diagnosis is possible only through a comprehensive assessment of the genetic resources preserved within these populations or species. Genetic diversity underpins a population’s ability to adapt to environmental changes, resist diseases and maintain overall fitness; understanding its extent and distribution is therefore paramount for devising effective conservation strategies [2].

Haplotype richness, defined as the number of distinct haplotypes present within a population, serves as a key indicator of genetic diversity [3]. High haplotype richness suggests a robust genetic reservoir, enhancing a population’s ability to respond to environmental challenges. Conversely, low haplotype richness may signal genetic bottlenecks, inbreeding or other factors that compromise viability [4]. However, observed haplotype counts frequently underestimate true richness due to sampling limitations. This limitation can be particularly acute in species with small, fragmented populations, elusive behaviour, and habitats that are difficult to access [5]. Predictive models and statistical approaches are therefore needed to extrapolate beyond observed data and to estimate the unseen fraction of genetic variation and better gauge a population’s genetic health [6,7].

The European mink *Mustela lutreola* exemplifies these challenges. Once widespread across Europe, the species has experienced dramatic contractions in range and abundance and is now classified as critically endangered [8]. Its current distribution is restricted to small, isolated populations in north-eastern (Russia and the captive stock of the European Association of Zoos and Aquaria (EAZA) Ex-situ Programme/EEP), south-eastern (Romania and Ukraine), and western Europe (France and Spain) [9]. Declines have been driven by habitat loss and degradation, overhunting, and competition with the invasive American mink *Neogale vison* [8]. Its cryptic behaviour and riparian habitat preferences further complicate monitoring and sampling, leaving major uncertainties about its distributional status and genetic resources [10].

Mitochondrial DNA (mtDNA) has long been a cornerstone of conservation genetics due to its maternal inheritance, high mutation rate, and the generally rare occurrence of recombination [11]. For *M. lutreola*, genetic studies have largely relied on portions of mtDNA sequences, primarily the hypervariable control region and cytochrome *b*, to infer diversity and differentiation [4,12]. Despite being informative, such analyses capture only a fraction of mitochondrial variation and risk underestimating haplotype richness. Advances in sequencing technologies have now enabled complete mitochondrial genomes (mitogenomes) to be obtained, offering a far more comprehensive view of variation [5].

Advances in high-throughput sequencing now allow the acquisition of complete mitochondrial genomes (mitogenomes), which provide a comprehensive view of mitochondrial variation and enable the detection of rare haplotypes often missed when only partial sequences are analysed [5]. Analyses of complete mitogenomes in other mustelids, including the fisher *Pekania pennanti* [13], Eurasian otter *Lutra lutra* [14], and European polecat *Mustela putorius* [15], have revealed previously unrecognised haplotypes, resolved phylogeographic structures, and informed conservation priorities. Comparable analyses for the European mink are still rare, but initial findings indicate that the species harbours more extensive diversity than previously suggested [5].

Given the limitations of observed haplotype counts and partial datasets, estimating the expected haplotype richness in *M. lutreola* requires predictive statistical tools. As a non-extrapolative measure, Fisher’s *α* derived from the log-series distribution provides a descriptive index of genetic diversity that is directly comparable across datasets of different sizes [16]. To correct for undersampling bias, non-parametric estimators such as *Jackknife1* [17], the *Bootstrap* estimator [18], and the abundance-based coverage estimator (*ACE*) [19] have been widely applied, each offering different sensitivities to the influence of rare haplotypes. A central concept in this framework is sample coverage (*SC*), the proportion of the total haplotype pool represented in a sample, which can be estimated directly and used to assess completeness [6]. Coverage-based rarefaction and extrapolation methods extend these approaches by relating diversity to sample completeness [7], while asymptotic estimators such as *Chao1* incorporate information from singletons and doubletons to infer unseen haplotypes [20]. In addition, when haplotype frequency distributions are highly overdispersed, as is often the case in small, fragmented populations, negative binomial models can be used to estimate the sample size required to detect a given proportion of haplotype richness [21]. Because these models rely on distributional assumptions, they are treated here as a complementary tool to gauge sampling effort rather than as a primary estimator.

The present study applies these approaches to complete mitogenome datasets of the European mink to estimate expected haplotype richness and sampling coverage across the western, south-eastern, and north-eastern populations. Building on the predictive framework introduced by Skorupski et al. [5], it is hypothesised that actual haplotype richness is considerably greater than suggested by observed data, and that substantial fractions of genetic variation remain unsampled. By analysing full mitogenomes, the study aims to uncover rare haplotypes and fine-scale structure that partial sequences cannot detect. The resulting estimates are intended to inform conservation by identifying populations with particularly depleted or diverse maternal lineages, thereby guiding captive breeding, reintroduction planning and genetic management to preserve the evolutionary potential of the European mink.

## 2. Results

### 2.1. Observed and Estimated Haplotype Richness

Analysis of 66 complete mitochondrial genomes yielded 41 unique haplotypes across the four extant populations of the European mink (Table S1). Observed haplotype richness ranged from six in Russia to thirteen in the France-Spain population (Table 1). The *ratio* of observed haplotypes per examined individual differed among populations, ranging from 0.87 in France–Spain and 0.75 in Romania to 0.55 in Russia and 0.42 in Germany. For the pooled European dataset, the ratio amounted to 0.62. Singletons accounted for 50% of Russian haplotypes, 75% of Romanian haplotypes, 60% of German haplotypes and 85% of French–Spanish haplotypes (Table 1).

Fisher’s *α*, calculated as a descriptive diversity index under the log-series model, was 5.40 for Russia, 21.81 for Romania, 6.44 for Germany, and 46.48 for France–Spain; the pooled dataset yielded *α* = 46.22. These values provide a non-extrapolative measure of haplotype diversity that reflects the balance between the number of haplotypes and individuals.

Non-parametric estimators indicated that the number of haplotypes present in each population is likely higher than the observed counts suggest (Table 1). Incidence-based *Jackknife1* returned moderate estimates (8.7 for Russia, 20.4 for Romania, 15.8 for Germany, and 23.3 for France–Spain; 69.6 for all populations combined), whereas the *Bootstrap* estimator yielded more conservative values, close to observed richness (7.2 for Russia, 15.5 for Romania, 12.4 for Germany, 17.1 for France–Spain; 52.7 combined). The abundance-based coverage estimator (*ACE*), which is especially sensitive to large numbers of singletons, produced higher estimates in populations dominated by rare haplotypes, particularly in France–Spain (48.8) and Romania (30.4), and in the pooled dataset (90.7).

Estimates from *Chao1*-based iNEXT asymptotic indicator were broadly consistent with these results, though wide confidence intervals were obtained, particularly in the western (France–Spain) and southeastern (Romania) populations (Table 1).

**Figure 1:**
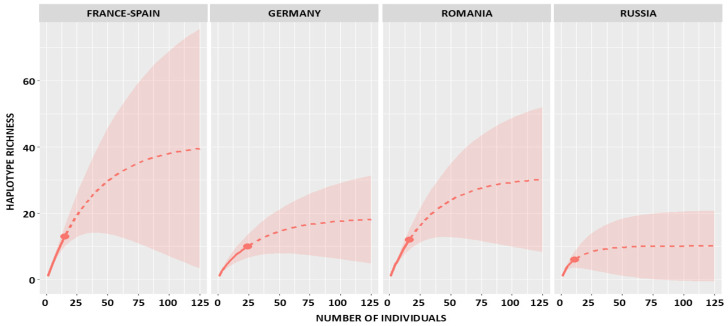
iNEXT rarefaction and extrapolation curves of haplotype richness in four populations of the European mink, with extrapolated values shown up to a standardized sample size of 125 individuals (solid lines represent interpolation, dashed lines extrapolation, and shaded areas represent 95% confidence intervals).

Rarefaction/extrapolation curves showed that haplotype richness increased steeply with the first few sampled individuals, reflecting the rapid accumulation of unique haplotypes, and then gradually levelled off (Figure 1). Extrapolation to a standardized sample size of 125 individuals indicated that true richness was considerably higher than the directly observed values. At this sampling depth, iNEXT estimated 10.09 haplotypes (95% CI: 1.02–19.15) for Russia, 30.20 (3.28–57.11) for Romania, 18.14 (0–37.29) for Germany, and 39.55 (10.87–68.23) for France–Spain. For the pooled European dataset, richness at *n* = 125 was estimated at 58.22 haplotypes, with comparatively narrow confidence intervals (95% CI: 50.87–63.93). Among individual populations, the western France–Spain population showed the highest projected haplotype richness.

**Figure 2:**
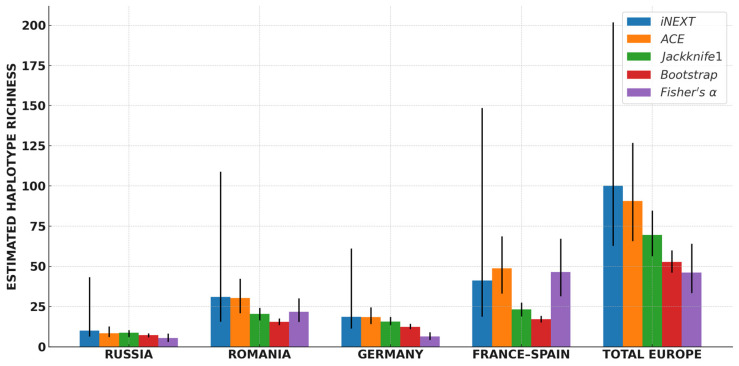
Comparison of haplotype richness estimates in four populations and the pooled European dataset of the European mink (vertical bars indicate 95% confidence intervals).

The comparison of non-parametric estimators indicated that all indices predicted higher haplotype richness than the observed values, albeit with marked differences among methods (Figure 2). The western France–Spain population consistently exhibited the highest estimated richness, followed by Romania, whereas Russia and Germany yielded lower values. Bootstrap produced the most conservative estimates, remaining close to observed richness, while ACE and iNEXT indicated substantially higher values in populations dominated by rare haplotypes (France–Spain, Romania). Jackknife1 provided intermediate results. Fisher’s *α*, as a descriptive non-extrapolative index, further emphasized contrasts among populations, with particularly elevated values in France–Spain.

### 2.2. Sampling Completeness and Coverage

At the observed sample sizes, Good–Turing coverage estimates indicated low completeness, with sampling coverage (*SC*) ranging from 0.29 in France–Spain to 0.76 in Germany, and 0.56 overall. The corresponding coverage deficits (1-*SC*) indicated that approximately 26% of haplotypes remain unsampled in Germany, 26% in Russia, 55% in Romania, 71% in France–Spain, and 44% across the pooled dataset (Table 1).

**Figure 3:**
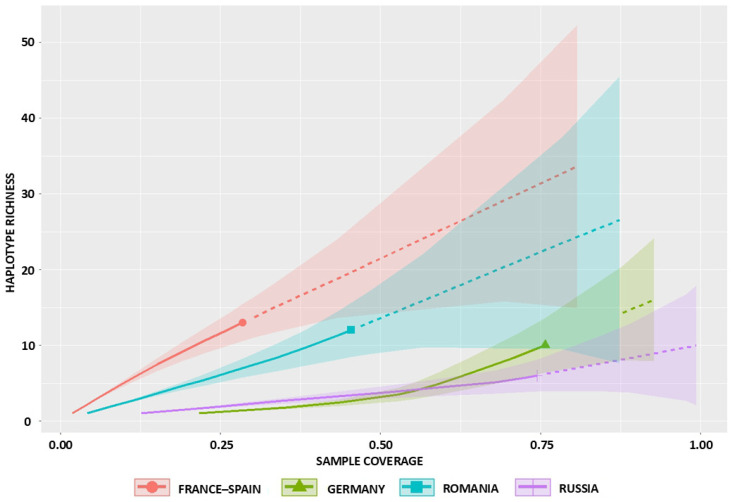
Rarefaction and extrapolation of haplotype richness as a function of sample coverage in four populations of the European mink, generated with iNEXT (solid lines represent interpolation, dashed lines extrapolation, and shaded areas indicate 95% confidence intervals).

Plots of haplotype richness versus sample coverage for the four populations (Figure 3) showed that the present datasets capture only a fraction of existing diversity, with coverage generally between 25% and 60% at current sample sizes. The lowest coverage was observed in the western France–Spain population, where about 30% of haplotypes were represented by 15 mitogenomes. In Germany, coverage was slightly higher (approximately 40–50%), reflecting a skewed distribution dominated by a single haplotype (45% of individuals). Romania and Russia both showed intermediate coverage of around 50%.

**Figure 4:**
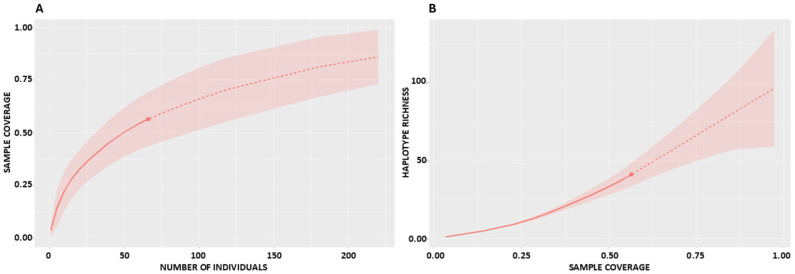
Rarefaction and extrapolation of haplotype richness in the pooled European dataset of the European mink, generated with iNEXT (solid lines represent interpolation, dashed lines extrapolation, and shaded areas indicate 95 percent confidence intervals). (**A**) Sample coverage as a function of the number of sampled individuals. (**B**) Relationship between haplotype richness and sample coverage.

Extrapolation to a standardized sample of 125 individuals demonstrated that coverage would increase substantially. At this level, Russia was predicted to reach complete coverage (*SC* = 1.0), Romania 0.977, Germany 0.986, and France–Spain 0.957. At the continental scale, coverage-based curves for the pooled dataset showed a steep initial rise, with observed coverage close to 55 percent at about 60 sequences and projected values approaching 90 percent at about 200 sequences (Figure 4A). Together with the per-population projections, these patterns indicate that sampling on the order of 100–130 sequences per population would capture most haplotypes, while several hundred sequences may be required to approach saturation for the pooled European dataset. The complementary analysis of haplotype richness as a function of sample coverage further emphasized the gap between observed and asymptotic diversity, with wide confidence intervals reflecting the influence of rare haplotypes (Figure 4B).

### 2.3. Required Sample Sizes

Negative binomial modelling was used to estimate the minimum number of sequences required to achieve a 95% probability of detecting all haplotypes in each population. The resulting sample size requirements varied markedly among populations, reflecting differences in haplotype frequency distributions. In Russia, where the sample already included several common haplotypes alongside a few rare variants, approximately 70 sequences would be sufficient to reach the 95% threshold. Romania required a slightly larger sample of about 80 individuals, owing to the predominance of singletons and a relatively even distribution of haplotype frequencies. The France–Spain population was estimated to require around 75 sequences to capture most haplotypes, consistent with its high asymptotic richness and large number of singletons. The German population exhibited the highest sampling demand, with an estimated 130 individuals needed, due to the strong dominance of a single haplotype, which comprised nearly half of the observed sequences.

Monte Carlo simulations indicated that these negative binomial-based estimates consistently achieved ≥95% capture probabilities. Furthermore, extrapolated coverage and richness values at a standardized sample size of 125 individuals corresponded closely to the predicted values. In particular, coverage values close to one at *n* = 125 in Russia and Germany suggest that sampling intensities on the order of 70–130 sequences per population would be adequate to recover the vast majority of haplotypes.

## 3. Discussion

This study provides the most comprehensive assessment to date of mitochondrial haplotype richness in the critically endangered European mink. By applying a suite of non-parametric and asymptotic estimators (*iNEXT*, *ACE*, *Jackknife1*, *Bootstrap*, and Fisher’s *α*), it was demonstrated that observed haplotype richness substantially underestimates true genetic diversity. This result is consistent with earlier genetic studies which noted that partial mitochondrial sequences and limited sampling capture only a fraction of the species’ genetic diversity. Findings from a meta-analysis of European mink studies, conducted by Skorupski et al. [5], detected significant gaps between measured and predicted haplotype diversity and variable sample coverage across populations—lowest in the Northeastern group (87%), moderate in Southeastern (96%), and highest in Western populations (99%).

In order to accurately predict true haplotype richness from observed data, a wide range of statistical estimators has been developed or adapted from biodiversity and statistical inference frameworks. Among the most widely used non-parametric estimators, *Chao1* and *Chao2* were explicitly designed to infer the number of unobserved haplotypes by exploiting the frequency of rare types (singletons and doubletons) in abundance and incidence data, respectively [22,23]. *Jackknife1* and *Jackknife2*, although originally proposed for species richness estimation, have also been applied to haplotype datasets, using resampling procedures to correct for undersampling bias [17]. The Good–Turing estimator, first developed for predicting unseen word frequencies [24], has been adapted to estimate the probability mass of undetected haplotypes, while the *Bootstrap* method provides a general resampling framework that can quantify uncertainty in richness predictions [25].

Other approaches have been tailored for datasets with a high proportion of rare haplotypes, including Zar’s method [26] and the Abundance-based Coverage Estimator (*ACE*) [19]. More advanced estimators include the Chao–Bunge model, which incorporates both rare and abundant haplotypes [27], and the Michaelis–Menten asymptotic model, which was adapted from enzymatic kinetics to describe haplotype accumulation curves [28]. Fisher’s *α*, derived from the log-series distribution, provides a robust index of diversity that is particularly informative for datasets characterised by uneven haplotype distributions [16]. The negative binomial distribution has also been used to model haplotype frequencies, offering a flexible framework for accounting for overdispersion and unobserved diversity [27,29]. In addition, non-parametric maximum likelihood estimation (*NPMLE*) [30], Hill numbers (*q*-diversity) [7,31], and Bayesian approaches employing Markov Chain Monte Carlo (*MCMC*) algorithms [32,33] have been applied to richness estimation, explicitly incorporating uncertainty or integrating richness with broader measures of diversity. Together, these estimators, whether originally conceived for haplotype richness or subsequently adapted, provide a robust and flexible foundation for predicting true genetic diversity, thereby supporting more accurate assessments critical for conservation management and evolutionary research.

Not all of these estimators were equally suitable for the present dataset. *Chao2*, for example, requires incidence data across multiple sites, which were not available in this study [23]. Similarly, more complex Bayesian *MCMC*-based methods demand large datasets and strong prior assumptions, which would be difficult to justify given the limited sample sizes available for European mink populations [32,33]. In contrast, estimators such as *Chao1* [22], *ACE* [19], *Jackknife1* [17], *Bootstrap* [18], Fisher’s *α* [16], and coverage-based rarefaction/extrapolation implemented in iNEXT [6,34] provided robust and interpretable results while accommodating the constraints of small, unevenly sampled populations. The application of the negative binomial model was further justified by the clear evidence of overdispersion in haplotype frequency distributions, particularly in Germany and Russia [21,35]. Together, this suite of complementary estimators allowed for a reliable characterisation of haplotype richness in the European mink, balancing statistical robustness with the practical limitations of available data.

The comparison of estimators revealed systematic differences. *Bootstrap* provided conservative values close to observed richness, *Jackknife1* produced intermediate estimates, while ACE and *iNEXT* yielded higher predictions in populations dominated by rare haplotypes. The sensitivity of *ACE* to singletons and doubletons explains its elevated estimates in France–Spain and Romania. Fisher’s *α*, although non-extrapolative, provided a useful descriptive measure, capturing relative richness independent of sample size. These complementary approaches illustrate the importance of applying multiple estimators when assessing genetic diversity in rare species [6,21]. The reliance on coverage-based rarefaction and extrapolation, as implemented in iNEXT, reflects current recommendations that standardisation by sample completeness is more robust than comparisons by sample size [6,36].

Extrapolation of sample coverage curves and negative binomial modelling indicated that between 70 and 130 sequences per population are required to achieve a 95% probability of capturing all haplotypes. This prediction is consistent with theoretical expectations, as haplotype richness typically rises steeply with the first sampled individuals before levelling off [20]. Validation through Monte Carlo simulation further confirmed the adequacy of these thresholds. These findings provide clear guidance for sampling design in conservation genetics: suboptimal sampling efforts risk severe underestimation of diversity and omission of rare haplotypes, which can disproportionately contribute to long-term evolutionary potential [35,37].

Marked differences in haplotype richness were observed among populations. The western France–Spain group consistently exhibited the highest observed and estimated richness across all estimators, whereas Romania showed moderately high richness but was dominated by singletons, leading to wide confidence intervals in extrapolated estimates. Russia and Germany harboured fewer haplotypes, with the German sample particularly affected by dominance of a single haplotype, producing low coverage and strong overdispersion. These contrasts align with expectations for small, fragmented populations in which genetic drift and stochastic loss of haplotypes shape diversity patterns [8,38]. Previous mitochondrial and microsatellite studies likewise reported high genetic diversity in north-eastern European populations and substantially lower diversity in western populations [4,12,39]. Those studies also highlighted strong regional differentiation, identifying three major populations (north-eastern, south-eastern and western Europe) and attributing reduced diversity in the western population to historical bottlenecks and limited gene flow.

Interpretation of these patterns requires caution, as the German dataset analysed in this study represents individuals from the EEP captive stock, which was founded from East European (Russian) individuals, whereas the Russian dataset also analysed here comprised individuals from the European Mink Breeding Centre in the Ilmen Nature Reserve [10,40]. Thus, both the German and Russian populations are derived from ex situ stocks and may not fully reflect the genetic composition of wild source populations. Such results remain consistent with long-recognised processes of population fragmentation and genetic drift, as emphasised in earlier reviews of European mink population genetics [9].

Previous mtDNA (D-loop, cyt *b*) and microsatellite studies likewise revealed strong regional differences, with the Western population often characterised by extreme homogeneity (sometimes a single haplotype detected), moderate diversity in the Southeast, and comparatively higher levels in the Northeast [4,12,39]. These studies also identified three genetically distinct populations (Northeastern, Southeastern, Western) with significant fixation index values indicating marked differentiation [4,39]. More recent syntheses emphasised that apparent low diversity in some populations may reflect incomplete sampling and short mtDNA markers, with rare haplotypes likely underdetected [5]. The present mitogenome-based results revise this picture, showing that the France–Spain population, previously considered genetically homogeneous, actually harbours the highest haplotype richness, whereas Romania also retains elevated richness but includes a large proportion of singletons, which increase the statistical uncertainty of the estimates. The present mitogenome-based results, which consistently recover higher predicted richness than previously observed, reduce the discrepancy between historical reports of extreme homogeneity in Western populations and the broader evidence of substantial mitochondrial variation. Thus, while earlier marker-based studies suggested a sharp contrast between Western and Southeastern groups, complete mitogenomes reveal that both retain valuable diversity and must be considered conservation priorities. These findings refine earlier conclusions by showing that, although demographic bottlenecks, restricted gene flow, and human-mediated fragmentation have shaped the current distribution of diversity in *M. lutreola*, considerable mitochondrial variation still persists within and among populations [9,10].

The observed heterogeneity among populations carries important implications for conservation management of *M. lutreola*. Populations with higher richness (France–Spain, Romania) represent key reservoirs of genetic diversity, whereas those with lower richness (Germany, Russia) appear more vulnerable to further loss and may require reinforcement or genetic rescue measures [1]. At the continental scale, the pooled dataset confirmed that significant diversity remains, underscoring the need for integrative management that preserves all extant populations. These results align with broader conservation genetic theory, which stresses the preservation of adaptive potential across fragmented groups and highlights the risks of inaction in genetically depauperate populations [1,41].

Several limitations of this study must be acknowledged. First, the analysis relied exclusively on mitochondrial DNA, which, while highly informative for maternal lineages, represents only a fraction of the total genetic diversity. Nuclear genomic data, such as whole-genome sequences, would provide a more complete picture of adaptive variation and demographic history [1,8]. Second, sample sizes varied among populations, with smaller datasets (e.g., Russia) producing wide confidence intervals and potentially inflating the influence of rare haplotypes. Although coverage-based extrapolation partially addresses this limitation, uneven sampling may still bias comparative estimates [6]. Finally, the estimators employed here, including *ACE*, *Jackknife1*, and negative binomial modelling, are sensitive to assumptions about the distribution of rare haplotypes. Integrating multiple approaches and validating results through simulation helped mitigate these uncertainties, but future studies should expand sampling to underrepresented populations and employ genomic-scale methods to refine haplotype discovery.

The recent publication of a platinum-standard reference genome for European mink provides a crucial resource for advancing conservation genomics [42]. This opens the door for integrating nuclear genomic datasets with mitochondrial analyses, enabling comprehensive assessments of adaptive potential and demographic history, particularly in genetically depauperate and isolated populations.

## 4. Materials and Methods

### 4.1. Sampling and Sequencing

**Figure 5:**
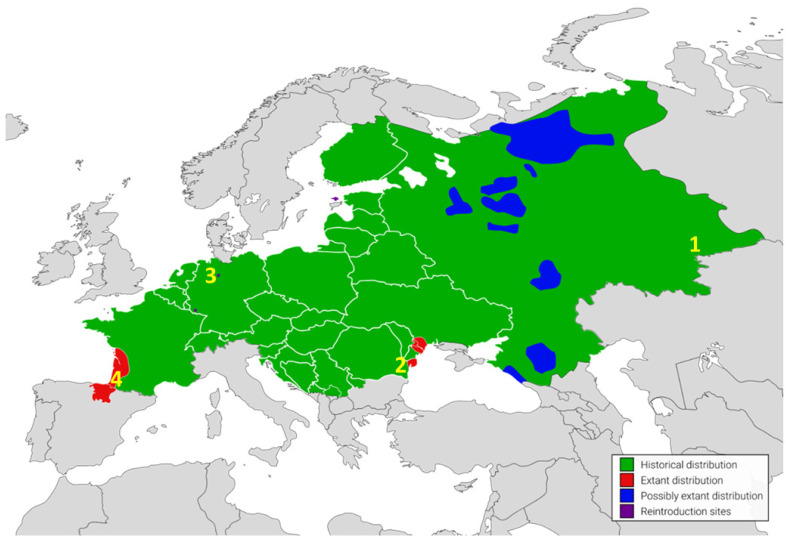
Historical and current range of the European mink *Mustela lutreola* in Europe. Numbers indicate the origin of mitogenomes analysed in this study: 1—Russia (Ilmen State Reserve and the Zoological Garden in Novosibirsk), 2—Romania (Danube Delta), 3—Germany (Zoological Garden in Osnabrück), 4—France–Spain (Charente, Gironde, Charente-Maritime, Landes, Pyrénées-Atlantique, Navarra).

A total of 66 complete mitochondrial genomes of the European mink were analysed, representing the four remaining populations: Russia (*n* = 11), Romania (*n* = 16), Germany (*n* = 24), France–Spain (*n* = 15) (Figure 5). All mitogenome sequences were retrieved from the GenBank genetic sequence database (https://www.ncbi.nlm.nih.gov/genbank/ (accessed on 30 June 2025; Tables S1 and S2). Haplotypes were defined as unique mitogenome sequences. To detect variable sites, all sequences were aligned using the Clustal Omega Multiple Sequence Alignment v. 1.2.4 software [43].

The complete mitochondrial genome of the European mink is 16,504 bp in length and contains the typical vertebrate set of 13 protein-coding genes, 22 tRNA genes, two rRNA genes, and a control region (D-loop). The gene order and structure are identical to those of other mustelids. The control region exhibits a short tandem repeat motif (5′-ACACAT-3′), and sequence variation among haplotypes primarily involves single-nucleotide substitutions in both coding and non-coding regions, with no frameshift mutations detected [44].

### 4.2. Observed Richness and Frequency Spectrum

For each population and for the pooled dataset, the observed number of haplotypes (*h_o_*) was recorded. Haplotypes occurring in ≤10 individuals were classified as rare (*h_r_*), and those occurring in >10 individuals as abundant (*h_a_*). The frequency spectrum was further characterised by the number of singletons (*f*_1_, haplotypes occurring once) and doubletons (*f*_2_, haplotypes occurring twice), which were subsequently used in non-parametric estimators.

### 4.3. Diversity Estimators

Diversity was quantified using complementary approaches. Fisher’s *α* was calculated as a non-extrapolative index under the log-series model [16]. Non-parametric estimators included the abundance-based coverage estimator (ACE) [19], incidence-based Jackknife1 [17], and the Bootstrap estimator [18]. Asymptotic richness was estimated using the Chao1-based framework implemented in iNEXT R package v.3.0.0 [34], which employs rarefaction and extrapolation with Hill numbers [7,31].

Calculations of Jackknife1, Bootstrap, ACE, sample coverage (*SC*), and Fisher’s *α* were performed in Python 3.11.13 [45] using custom scripts, with implementations based on the original published formulas. Statistical validation of these indices (10,000 bootstrap replications, split-half reliability, fixed-effort rarefaction repeats) was conducted using NumPy v.1.26.4 [46], pandas v.2.2.2 [47], and SciPy v.1.13.1 [48].

### 4.4. Sample Coverage

Sample coverage (*SC*), defined as the proportion of the total haplotype pool represented in the sample, was estimated using the Good–Turing frequency formula [24] with the bias-corrected form of Chao & Jost [6]. The complement (1-*SC*) was calculated as the coverage deficit, i.e., the probability that the next sampled individual would represent an undetected haplotype. *SC* values were calculated according to the original published formula, implemented in Python 3.11.13 [45], with non-parametric bootstrap validation (B = 10,000).

### 4.5. Rarefaction and Extrapolation

Coverage-based rarefaction and extrapolation curves were generated in the R package iNEXT v.3.0.0 [34], following the framework of Chao et al. [7]. Estimates were standardised to a common sample size of *n* = 125 individuals for inter-population comparison. Rarefaction/extrapolation was performed with 10,000 bootstrap replicates to generate 95% confidence intervals around richness and coverage estimates. All figures of rarefaction/extrapolation and coverage curves were produced in iNEXT, while a comparative bar chart of richness estimators was prepared in Matplotlib v.3.7.1 [49].

### 4.6. Sample Size Requirements

To estimate the sampling effort required to capture ≥95% of haplotype richness in each population, negative binomial models were fitted to haplotype frequency distributions [21]. Monte Carlo simulations (10,000 iterations) were used to verify that predicted sample sizes achieved the required coverage threshold [50].

Mean and variance of haplotype frequencies were calculated from the per-population frequency distributions, and variance-to-mean ratios (VMR) confirmed overdispersion in Germany (VMR = 5.55), Russia (VMR = 2.05), and the pooled European dataset (VMR = 1.67), with weaker but detectable overdispersion in Romania (VMR = 1.27). France–Spain showed nearly Poisson-like behaviour (VMR = 0.92). The confirmation of overdispersion justified the use of the negative binomial model, which is well suited for biological count data in which variance exceeds the mean [35].

Parameters of the negative binomial distribution were estimated using the method-of-moments approach, which equates observed mean and variance to theoretical distributional moments [51]. This parameterization accounts for heterogeneity in haplotype frequencies, particularly the influence of rare haplotypes such as singletons and doubletons. For each population, the minimum sample size was then estimated as the number of sequences required for the probability of detecting all observed haplotypes to exceed 95%. Simulation-based validation confirmed the robustness of these predictions.

All calculations were conducted in Python 3.11.13 [45] using NumPy v.1.26.4 [46] and SciPy v.1.13.1 [48] libraries.

## 5. Conclusions

Complete mitochondrial genome analyses provided a clearer and more comprehensive view of haplotype diversity in the European mink. Observed haplotype counts were consistently lower than those predicted by non-parametric estimators, indicating that substantial mitochondrial variation remains unsampled. While most estimators yielded consistent results, the width of confidence intervals, particularly in the France–Spain and Romanian populations, reflects the strong influence of rare haplotypes and limited sample coverage on richness uncertainty.

These findings refine earlier views of mitochondrial diversity, revealing considerable variation across the species’ range and challenging assumptions of homogeneity in the western population. Genetic structuring among Western, Southeastern and Northeastern groups supports their treatment as distinct conservation units, with reinforcement restricted to the same lineage origin and conservation efforts prioritised for the more diverse France–Spain and Romanian populations. Genetically depleted Russian and German populations, both represented by ex situ lineages, may require genetic rescue through the addition of wild individuals from extant Eastern European populations. At the same time, the rapid ecological deterioration of habitats and populations may in future challenge strictly lineage-based management, requiring a careful balance between maintaining genetic integrity and preventing demographic collapse.

Sampling thresholds of approximately 70–130 individuals per population, as identified here, provide practical guidance for future genetic surveys to achieve near-complete mitogenome haplotype detection. While mitogenomes capture maternal lineage diversity, nuclear genomic data are required to assess adaptive potential, inbreeding and demographic processes. Integrating these insights into ex situ programmes and in situ management is essential to safeguard the evolutionary potential of this critically endangered species.

## Figures and Tables

**Table 1:** Observed and estimated haplotype richness in four populations of *M. lutreola*.

Population	*n*	*h_o_*	*h_r_*	*h_a_*	*n_r_*	*f*_1_	*f*_2_	iNEXT (*CI*_0.95_)	*ACE* (*CI*_0.95_)	*Jackknife1* (*CI*_0.95_)	*Bootstrap* (*CI*_0.95_)	*Fisher’s α* (*CI*_0.95_)	*SC* (*CI*_0.95_)
Russia	11	6	6	0	11	3	1	10.09 (6.45–43.40)	8.46 (6.20–12.8)	8.73 (6.10–10.50)	7.22 (6.00–8.40)	5.40 (3.10–8.20)	0.74 (0.64–1.00)
Romania	16	12	12	0	16	9	2	30.98 (15.72–108.90)	30.37 (21.00–42.30)	20.44 (16.50–24.30)	15.48 (13.50–17.60)	21.81 (15.50–30.20)	0.45 (0.31–0.66)
Germany	24	10	9	1	13	6	2	18.63 (11.45–61.17)	18.51 (14.20–24.60)	15.75 (13.50–18.60)	12.45 (11.00–14.30)	6.44 (4.40–9.10)	0.76 (0.58–0.93)
France-Spain	15	13	13	0	15	11	2	41.23 (18.88–148.66)	48.75 (33.20–68.80)	23.27 (19.00–27.50)	17.14 (15.20–19.30)	46.48 (31.50–67.20)	0.29 (0.18–0.39)
TOTAL	66	41	40	1	55	29	7	100.16 (62.75–201.90)	90.68 (65.70–126.80)	69.56 (56.40–84.70)	52.69 (46.10–60.00)	46.22 (33.40–64.10)	0.56 (0.47–0.65)

*n*—sample size, *h_o_*—observed haplotypes, *h_r_*—rare (≤10 individuals) haplotypes, *h_a_*—abundant (>10 individuals) haplotypes, *n_r_*—total count of individuals carrying rare haplotypes, *f*_1_—number of haplotypes occurring once (singletons), *f*_2_—number of haplotypes occurring twice (doubletons), *α*—Fisher’s diversity index (non-extrapolative), *SC*—sample coverage, *CI*_0.95_—95% confidence interval.

## Data Availability

The original data presented in the study are openly available in the GenBank^®^ genetic sequence database at https://www.ncbi.nlm.nih.gov/genbank/.

## Supplementary Materials

The following supporting information can be downloaded at https://www.mdpi.com/article/10.3390/ijms26209935/s1.
