# Supplementary material for: Expected Mitochondrial Haplotype Richness in Remaining Populations of the Critically Endangered European Mink Mustela lutreola and Its Conservation Implications
Source: Int J Mol Sci. 2025 Oct 12;26(20):9935. doi: 10.3390/ijms26209935 (PMC12563210; doi:10.3390/ijms26209935)
Supplement: Supplementary file 1 [file ijms-26-09935-s001.zip › Supplementary/Supplementary_-_Table_S2.pdf]

Table S2. Characteristics of European mink samples used in the study

Individual ID	n	Collection year	Origin		GenBank Accession No.
RU1	11	2018	Russia	Ilmen State Reserve SU FRC MG, Ural Branch of the Russian Academy of Science (Miass, Chelyabinsk Oblast)	OQ570453
RU2		2018			OQ570452
RU3		2018			OQ570439
RU4		2018			OQ570438
RU5		2018			OQ570451
RU6		2018			OQ570450
RU7		2018			OQ570448
RU8		2018			OQ570458
RU9		2018			OQ570440
RU10		2018		The Zoological Garden in Novosibirsk (Novosibirsk, Novosibirsk Oblast)	OQ570447
RU11		2018			OQ570437
RO1	16	2018	Romania	The "Danube Delta" National Institute for Research and Development (Tulcea, Tulcea County)	OQ570428
RO2		2018			OQ570426
RO3		2021			PQ826902
RO4		2021			PQ826903
RO5		2022			PQ826904
RO6		2023			PQ826905
RO7		2023			PQ826906
RO8		2022			PQ826907
RO9		2022			PQ826908
RO10		2023			PQ826909
RO11		2022			PQ8269010
RO12		2022			PQ826911
RO13		2024			PQ826912
RO14		2022			PQ826913
RO15		2023			PQ826914
RO16		2023			PQ826915
DE1	24	2011	Germany	Zoological Garden in Osnabrück (Osnabrück, Lower Saxony)	OQ570432
DE2		2011			OQ570459
DE3		2012			OQ570425
DE4		2012			OQ570462
DE5		2012			NC_056132
DE6		2012			OQ570454
DE7		2013			MT304869
DE8		2014			OQ570424
DE9		2014			OQ570460
DE10		2014			OQ570461
DE11		2014			MW197423
DE12		2012			OQ570465
DE13		2012			OQ570464

DE14		2012			OQ570456
DE15		2012			MW197424
DE16		2013			OQ570457
DE17		2013			OQ570466
DE18		2013			OQ570446
DE19		2014			OQ570430
DE20		2014			MW197425
DE21		2014			OQ570468
DE22		2014			MW197426
DE23		2014			OQ570455
DE24		2015			OQ570467
FR1	15	2002	France	Charente	OQ570444
FR2		2002		Charente	OQ570449
FR3		2002		Gironde	OQ570429
FR4		2003		Charente	OQ570445
FR5		2003		Charente-Maritime	OQ570427
FR6		2018		Charente-Maritime	OQ570434
FR7		2001		Gironde	OQ570442
FR8		2001		Charente	OQ570443
FR9		2001		Landes	OQ570435
FR10		2002		Landes	OQ570469
FR11		2002		Pyrénées-Atlantique	OQ570441
FR12		2003		Charente-Maritime	OQ570431
FR13		2003		Gironde	OQ570433
FR14		2018		Charente-Maritime	OQ570436
SP1		2017	Spain	Navarra	OQ570463
